# Putting Up a Big Front: Car Design and Size Affect Road-Crossing Behaviour

**DOI:** 10.1371/journal.pone.0159455

**Published:** 2016-07-19

**Authors:** Wilhelm K. Klatt, Alvin Chesham, Janek S. Lobmaier

**Affiliations:** 1 Institute of Psychology, University of Bern, Bern, Switzerland; 2 Center for Cognition, Learning and Memory, University of Bern, Bern, Switzerland; 3 ARTORG Center for Biomedical Engineering Research, Gerontechnology & Rehabilitation, University of Bern, Bern, Switzerland; University of Rome, ITALY

## Abstract

Previous research suggests that people tend to see faces in car fronts and that they attribute personality characteristics to car faces. In the present study we investigated whether car design influences pedestrian road-crossing behaviour. An immersive virtual reality environment with a zebra crossing scenario was used to determine a) whether the minimum accepted distance for crossing the street is larger for cars with a dominant appearance than for cars with a friendly appearance and b) whether the speed of dominant-looking cars is overestimated as compared to friendly-looking cars. Participants completed both tasks while either standing on the pavement or on the centre island. We found that people started to cross the road later in front of friendly-looking low-power cars compared to dominant-looking high-power cars, but only if the cars were relatively large in size. For small cars we found no effect of power. The speed of smaller cars was estimated to be higher compared to large cars (size-speed bias). Furthermore, there was an effect of starting position: From the centre island, participants entered the road significantly later (i. e. closer to the approaching car) and left the road later than when starting from the pavement. Similarly, the speed of the cars was estimated significantly lower when standing on the centre island compared to the pavement. To our knowledge, this is the first study to show that car fronts elicit responses on a behavioural level.

## Introduction

Human beings possess a highly developed sensitivity for facial features. From faces we gather information about sex, age, emotions and intentions of our counterpart. We also often attribute human characteristics even to non-human beings and inanimate objects. Such anthropomorphisms are interpreted as a result of an evolutionary error management strategy. Humans as social beings have a hyperactive agent-detection system because the cost of missing an agent is much higher than falsely detecting one [1–3]. As a result, we are lead to detect faces everywhere–even in inanimate objects, such as cars [1]. This illusory perception of non-existing faces is referred to as face pareidolia and leads to fusiform face area activation even when looking at pure-noise images [4]. Windhager et al. [5] experimentally demonstrated that about 90% of the participants perceived facial characteristics in car fronts. Participants were asked to compare specific features (eyes, nose, mouth, ears) of a face and a car front presented next to each other while eye movements were recorded. Eye movement patterns were highly similar when participants looked at faces and car fronts: When asked to compare the nose, there were twice as many fixations on the grille compared to the other features; similarly when asked to compare the ears, fixations were more than double as often on the side-view mirrors than on the remaining features. In line with existing findings in face perception, participants predominantly fixated the eyes and the headlights, even in tasks where they had to compare something else. Recent neurophysiological and imaging studies have provided further support for the notion that face-like objects and faces are processed in a similar fashion. For example, car fronts elicit similar neural activity as human faces in studies using EEG/ERP [6–9], MEG [10], and fMRI [11, 12].

Landwehr, McGill, and Herrmann [13] found that even emotional expressions can be attributed to car faces. They presented participants with car fronts of different models in which headlights (the supposed equivalent of the eyes) and grille (the supposed equivalent of the mouth) had been changed by professional car designers. Participants rated car fronts with upturned grilles friendlier, irrespective of the headlights. High ratings in aggressiveness resulted from slanted headlights in combination with a downturned grille.

Purucker, Sprott, and Herrmann [14] tested the reactions to the anthropomorphic design of car fronts in an approach-avoidance paradigm using eye tracking methodology. Several features of car fronts were manipulated to appear more or less threatening. They found that participants paid more attention to threatening car fronts than to non-threatening car fronts. Furthermore, participants tended to avoid the threatening car fronts compared to non-threatening ones.

Windhager, Slice, Schaefer, Oberzaucher, Thorstensen, and Grammer [15] investigated whether car faces of existing models contain information about personality characteristics and emotional expressions as do human faces. Participants were asked to rate 38 car models on 19 personality traits. A Principal Components Analysis revealed that variation in the perception of personality characteristics in car faces was explained essentially by one component (83% total variation). This factor with loadings on attributes such as “adult, dominant, arrogant, angry, masculine, hostile” was referred to as “power”. When asked to indicate the extent to which they perceived each car to have a face and to identify the facial features (eyes, nose, mouth, ears), more than 60% of the participants saw a face in at least 70% of the car fronts; facial features were marked with high agreement in all car models [15]. This finding was replicated in an Ethiopian sample, rendering cultural influences on car front perception unlikely [16].

How do the findings of Windhager et al. [15] translate to actual road traffic behaviour? In studies investigating road-crossing behaviour, various approaches have been used. In the *Shout* and *Two-step* tasks, participants wait at an actual roadside and indicate by calling or by stepping forward when they would cross the road. Demetre et al. [17] found that, compared to adults, children behaved in an overcautious way, resulting in a higher number of missed opportunities to cross the road. The disadvantage of these procedures is that they measure hypothetical decisions rather than actual behaviour. When effectively crossing a road, participants’ cognitive and behavioural responses may be restricted due to the physical dangers involved. There is also no experimental control over potentially important variables such as speed and distance of approaching vehicles [18]. Other approaches include observational and quasi-experimental field studies. Piff, Stancato, Côté, Mendoza-Denton, and Keltner [19] positioned a confederate at a zebra crossing in a busy street and recorded the scene with video cameras. Many drivers failed to stop at the crossing even though the pedestrian had right of way at this point. Interestingly, the more expensive a car, the less willing the driver was to stop. In the lowest-priced car category, not one single car ignored the pedestrian’s right of way, whereas nearly 50% of the drivers of upper-class cars cut off the pedestrian.

Other researchers have investigated road-crossing behaviour in the laboratory. For example, Pitcairn and Edlmann [20] asked participants to press a key when they would cross the street while video sequences showing a street were presented on a television screen. Children performed less well than adults (more delays, fewer safe crossings, higher number of missed opportunities) and showed larger individual differences. Another study investigated the accident risk of adolescents with and without attention deficit hyperactivity disorder (ADHD) in virtual reality [18]. Vans approached at different intervals and the task was to choose gaps in which it was maximally safe to cross the road. Adolescents with ADHD made fewer safe road-crossings, had a lower walking speed, used fewer gaps in traffic, exhibited a larger variability and had more than twice as many crashes than the control group without ADHD [18]. In this study, the design and the velocity of the vehicles was kept constant. In addition, from the perspective of the participant the vehicles approached from one direction only.

If car fronts are perceived in a similar way as human faces and receive attributions of personality characteristics, one could expect car design to have a bearing on the behaviour of other road users. Previous studies have not taken car design into account [18] or have differentiated only between vehicle categories such as car, truck and bus [21], saloon, sport utility vehicle and truck [22] or car and bus [23]. In the present study we specifically examine the influence of car design on the behaviour of pedestrians in a virtual environment. We chose a virtual reality setting because virtual reality enables realistic settings with high-quality sound and the ability to interact, making the participant feel present in that environment. Virtual reality allows participants to explore situations and to act in a safe setting, making it ideal for pedestrian experiments [22]. Finally, virtual reality makes it possible to keep various variables constant, which would be impossible in a field experiment: Weather, lighting conditions, volume of traffic, velocity of vehicles, colour of vehicles, noise, other pedestrians and appearance of the driver can be controlled for and can therefore be ruled out as interfering variables.

Participants stood at a pedestrian crossing in virtual reality and crossed the road in front of approaching cars. Car models were either dominant-looking cars (high-power cars) or cars with a friendly face (low-power cars, cf. Windhager et al. [15]). We predicted that people would cross the road earlier when a high-power car was approaching (i.e., when the car was still farther away) than when a low-power car was approaching. Because it is likely that participants make their decisions on the basis of speed estimations, we further asked participants to judge the speed of passing cars in a second experimental block. Here we expected that the speed of high-power cars would be overestimated compared to low-power cars.

## Method

### Participants

Sixty young healthy adults (30 female, 30 male) between 20 and 31 years of age (*M* = 23.1, *SD* = 2.1) took part in this study. All provided written informed consent to participate. This study was approved by the ethics committee of the Faculty of Human Sciences of the University of Bern (approval number: 2013-10-665553) and participants were treated in accordance with the Code of Ethics of the World Medical Association (Declaration of Helsinki).

### Apparatus

Participants viewed the virtual environment wearing an nVisor SX60 stereoscopic head-mounted display (HMD) with a resolution of 1280 × 1024 and a diagonal field of view of 60° in each eye. Head rotations were tracked using an InertiaCube3 three-axis orientation tracker attached to the head-mounted display. The position of the participant was tracked using PPT studio 2008 version 3.1. The orientation and position information was transferred to the rendering machine to update the graphics displayed in the HMD. The virtual zebra crossing scenario was scripted and rendered using WorldViz version 4.0 32-bit.

Real recordings of a BMW E28 driving by at the different speeds outlined below were played back with a Technics SU-V60 hi-fi amplifier and two Technics SB-M5 speakers. Volume was adjusted to 80 decibel, reflecting the realistic noise level of a car [24].

## Materials

### Virtual reality

**Fig 1:**
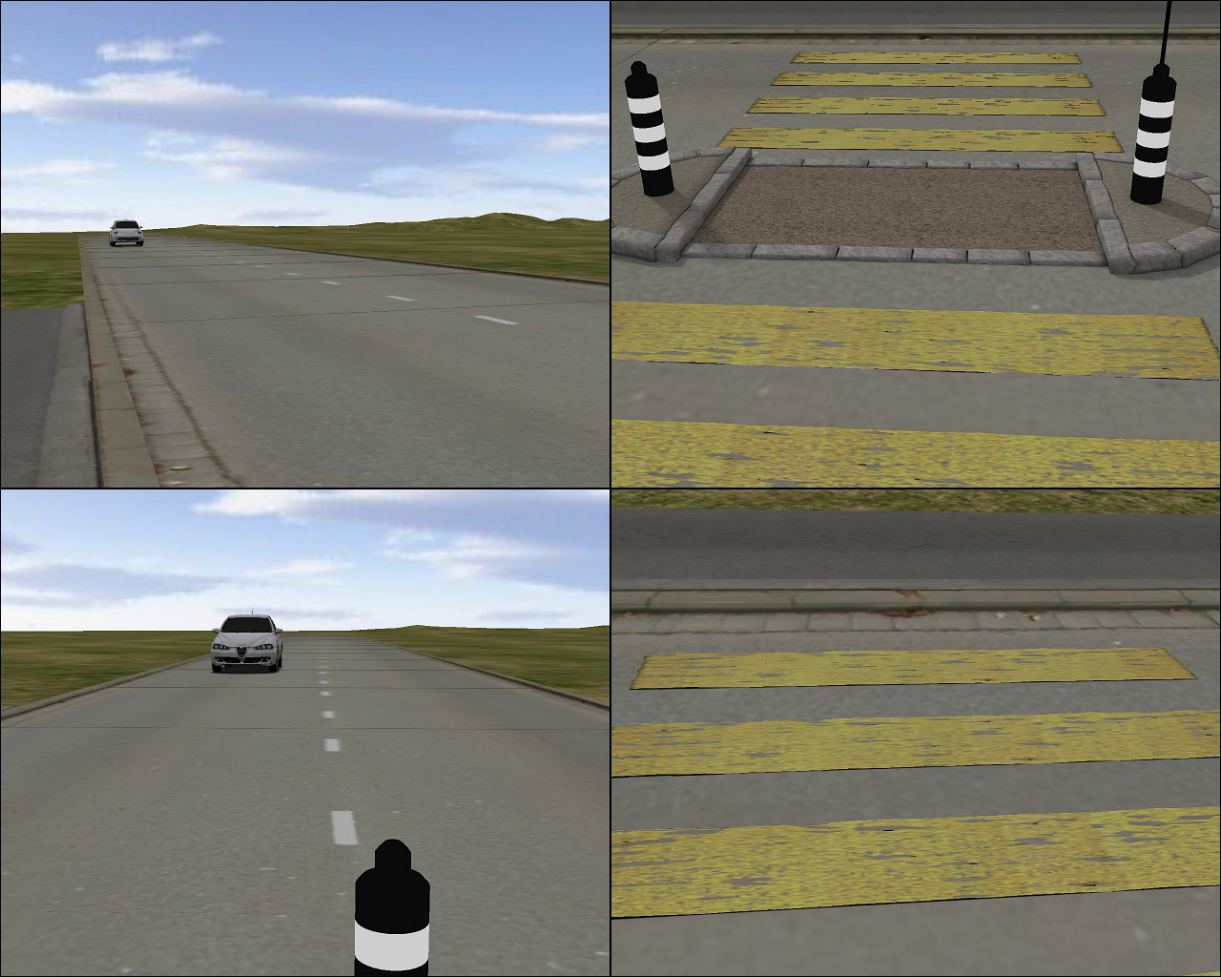
**The participants’ perspective standing on the pavement (top) and on the centre island (bottom)**.

The virtual environment consisted of a straight two-lane road of 130 metres in length and a pedestrian crossing with a traffic island. The road was surrounded by a hilly grassy landscape. The zebra crossing scenario was modelled, textured and baked using the open-source 3D graphic software Blender (Blender Foundation, 2015). From the participants’ point of view, the road was 100 metres long in the direction of the oncoming cars and ended after 30 metres in the other direction. The cars appeared individually at a distance of 100 metres and approached with a speed of 50 km/h (13.89 m/s) in the road-crossing block or with 45/50/55 km/h (12.5/13.89/15.28 m/s) in the speed-estimation block, passed the pedestrian crossing without stopping and disappeared at the end of the road (30 metres down). Fig 1 illustrates the virtual environment.

**Fig 2:**
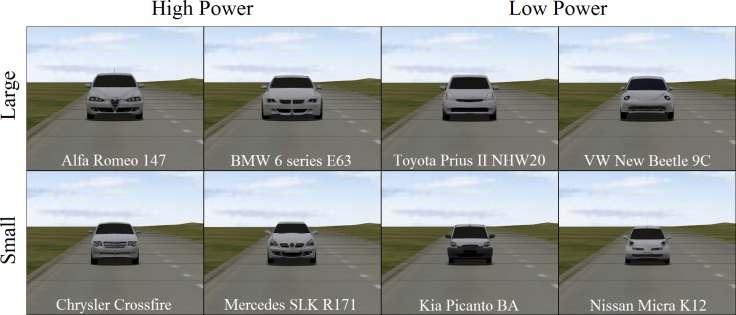
Vehicles used in virtual reality.

Stimuli were eight vehicles, four with a high-power appearance (rated as angry, dominant, hostile: BMW 6 series E63, Alfa Romeo 147, Mercedes SLK R171, Chrysler Crossfire) and four with a low-power appearance (rated as happy, open, childlike, agreeable: Nissan Micra K12, VW New Beetle 9C, Kia Picanto BA, Toyota Prius II NHW20) (cf. Windhager et al., 2008). Three-dimensional models of these cars were downloaded from open content 3D model repositories (http://www.dmi-3d.net/ and http://3dwarehouse.sketchup.com) and were reworked individually. The body and rims were coloured in light grey (RGB: 0.8, 0.8, 0.8) and textured using Voronoi (Noise size: 0.001) for a glossy and glitter effect of metallic paint. The window panes and the tires were coloured black (RGB: 0.0, 0.0, 0.0), while the remaining parts of the car (grilles, window frame, bumpers, window wipers etc.) were coloured grey (RGB: 0.15, 0.15, 0.15). For added realism, realtime cubemaps, reflecting the surrounding virtual environment, were blended onto the window panes, body of the car and rims to simulate surface reflection (see Fig 2). To control for size as a confounding factor, two of the high-power cars were relatively large models (Alfa Romeo, BMW) and two were relatively small (Chrysler, Mercedes) and two of the low-power cars were relatively large (Toyota, VW) and two were relatively small (Kia, Nissan). The size of the front surface of each car was measured in pixels using Adobe Photoshop CS6. A prior t-test showed no significant difference in the size (front surface) of low and high-power cars (*t*(3) = 0.76; *p* > .50).

To ensure that similar personality attributes were ascribed to these virtual cars as in Windhager et al.’s study [15], an independent sample of 44 participants rated screenshots of the cars for the same 18 personality characteristics as in Windhager et al. [15]. In line with their findings, high-power cars compared to low-power cars were rated as being more arrogant (*F*(1,43) = 126.67, *p* < .001, η_p_^2^ = .747), more extraverted *F*(1,43) = 19.334, *p* < .001, η_p_^2^ = .31, less surprised (*F*(1,43) = 22.424, *p* < .001, η_p_^2^ = .343), less afraid (*F*(1,43) = 81.46, *p* < .001, η_p_^2^ = .655), more angry (*F*(1,43) = 70.124, *p* < .001, η_p_^2^ = .62), less sad (*F*(1,43) = 25.915, *p* < .001, η_p_^2^ = .376), less agreeable (*F*(1,43) = 30.399, *p* < .001, η_p_^2^ = .414), more adult (*F*(1,43) = 105.466, *p* < .001, η_p_^2^ = .71), more dominant (*F*(1,43) = 185.696, *p* < .001, η_p_^2^ = .812), more hostile (*F*(1,43) = 75.233, *p* < .001, η_p_^2^ = .636), and more masculine (*F*(1,43) = 119.196, *p* < .001, η_p_^2^ = .735).

### Task and Procedure

On arrival, participants were informed of the procedure and provided written informed consent. Then the head-mounted display (HMD) was attached and the virtual setting for the road-crossing task (Block 1) was loaded. Before the experiment proper, participants were given the opportunity to explore the virtual environment and to familiarize themselves with the road and the pedestrian crossing. Participants were told from which direction the vehicles would appear and were instructed that they would be alternately crossing the road starting from the pavement or island. Participants were also told that the cars would be passing through without stopping and were asked to cross the road in front of an approaching car at the latest moment they considered safe. At the beginning of each trial, participants were told to look towards the end of the road in the direction of the oncoming cars. They were supervised carefully at all times; subsequent trials were only initiated if the participant was standing still at the new position and was ready to cross the road. Each of the eight cars was presented four times, resulting in 32 trials. The presentation order of the cars was randomized in both blocks. After the trials in Block 1 participants could take a short break. They were then given instructions for the speed-estimation task (Block 2). They were told that their task would be to estimate the speed of each passing car in kilometres per hour (km/h). Estimates were made verbally and fed into the computer by the experimenter. Only after an estimation was made did the experimenter start the next trial. Each car was presented three times but with different speeds (45, 50, 55 km/h) both when the participant was standing on the pavement and on the centre island, resulting in 48 trials.

Upon completion of the virtual-reality tasks participants were asked to answer a short online questionnaire. Based on Clancy et al. [18], participants were asked whether they suffered from ADHD, whether they had normal vision, whether they had ever observed or been involved in a pedestrian accident, whether they had a driving licence and how frequently they drove a car (cf. Hamed [23]). Other questions asked about car brand preferences, how often on average the participant crossed a street at a pedestrian crossing, handedness and drug use. Finally, we asked whether the participant suspected what the study was about. Participants were then debriefed and were thanked for participation. The entire experiment took approximately one hour to complete.

## Results

### Questionnaire Data

Two participants reported suffering from ADHD, eleven had observed and four had been involved in a pedestrian accident, 44 were frequent drivers and 54 were right-handed. For both blocks separately, an analysis of covariance was run with these variables. None of them explained a significant part of the variance (*F*(1,44) ≤ 1.894, all *p*’s > .10, η_p_^2^ ≤ .041), hence these variables were ignored in subsequent analyses.

### Road-crossing behaviour

A 2 (power) × 2 (car size) × 2 (starting position) repeated measures ANOVA was run separately for starting time, arrival time, and crossing duration using SPSS 22. Data of four participants were excluded from analysis because of technical problems during data collection.

#### Starting time

**Fig 3:**
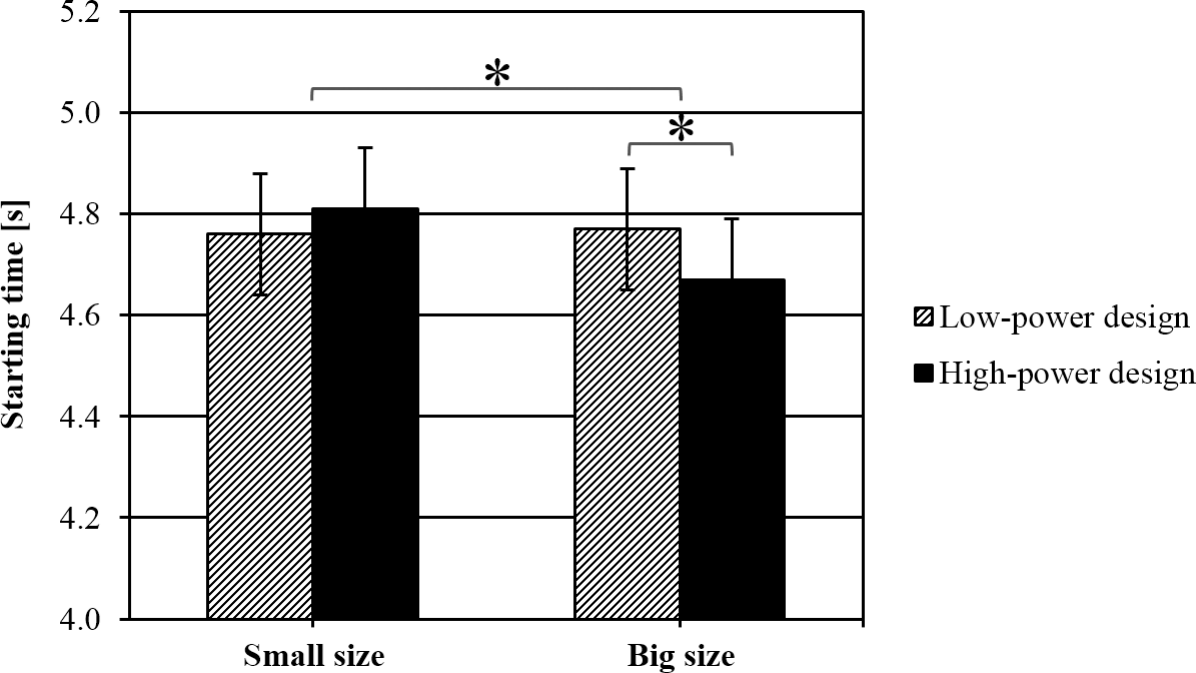
Starting time, depending on power and car size. Error bars represent standard errors.

Power alone did not affect the point in time participants chose to cross the road (*F*(1,55) = .277, *p* = .601, η_p_^2^ = .005) but there was a significant main effect of car size, *F*(1,55) = 6.622, *p* = .013, η_p_^2^ = .107. Participants started to cross the road significantly earlier in front of big (*M* = 4.72 s; *SD* = 0.99) compared to small cars (*M* = 4.80 s; *SD* = 0.96). Importantly, the power × car size interaction reached statistical significance (*F*(1,55) = 5.542, *p* = .022, η_p_^2^ = .092), suggesting an influence of car design only for big cars (see Fig 3). Participants started to cross the road significantly earlier in front of big high-power cars compared to big low-power cars, *t*(59) = 2.23; *p* = .029. For small cars there was no difference in starting time between high and low power (*t*(59) = -1.337; *p* = .19). No other interaction reached statistical significance (*F*(1,55) ≤ 1.276, all *p*’s > .26, η_p_^2^ ≤ .023). Surprisingly, there was a significant main effect of starting position (*F*(1,55) = 10.845, *p* = .002, η_p_^2^ = .165). Starting from the centre island, participants entered the road significantly later than when starting from the pavement.

#### Arrival time

Similar to the analysis of starting time, power did not affect the time of arrival on the other side of the road (*F*(1,52) = 0.077, *p* = .78, η_p_^2^ = .001), but there was a main effect of car size, *F*(1,52) = 10.232, *p* = .002, η_p_^2^ = .164. Participants arrived at the other side of the lane significantly earlier in front of large cars (*M* = 6.47 s; *SD* = 0.77) compared to small cars (*M* = 6.58 s; *SD* = 0.87). Again, the power × car size interaction was significant, *F*(1,52) = 6.563, *p* = .013, η_p_^2^ = .112. Follow-up t-tests revealed that participants arrived at the other side of the lane significantly earlier in front of large high-power cars compared to large low-power cars, *t*(59) = 2.374; *p* = .021. For small cars, the power had no significant effect on arrival time (*t*(59) = – 0.898; *p* = .37). Finally, there was a main effect of starting position (*F*(1,52) = 21.565, *p* < .001, η_p_^2^ = .293). Participants reached the other side earlier when starting from the pavement (*M* = 6.42 s; *SD* = 0.77) than when starting from the centre island (*M* = 6.63 s; *SD* = 0.84). No interaction except the reported power × size interaction reached statistical significance (*F*(1,52) ≤ 1.090, all *p*’s > .30, η_p_^2^ ≤ .021).

#### Walking speed

Power and car size did not have an effect on walking speed (power: *F*(1,51) = .263, *p* = .61, η_p_^2^ = .005; size: *F*(1,51) = .159, *p* = .692, η_p_^2^ = .003). The only significant effect was that of starting position (*F*(1,51) = 6.400, *p* = .015, η_p_^2^ = .112), participants crossed the road significantly faster when starting from the pavement (*M* = 1.72 s; *SD* = 0.43) compared to starting from the centre island (*M* = 1.80 s; *SD* = 0.43). No interaction reached statistical significance (*F*(1,51) ≤ .381, all *p*’s > .54, η_p_^2^ ≤ .007).

### Speed estimations

**Fig 4:**
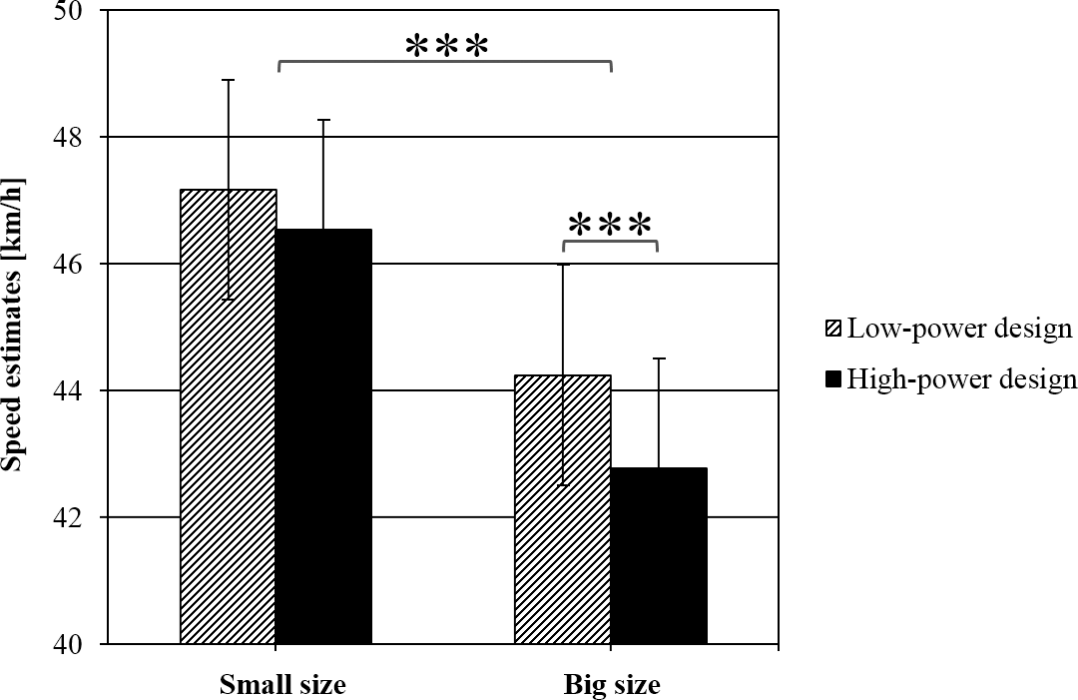
Speed estimations, depending on power and car size. Error bars represent standard errors.

**Fig 5:**
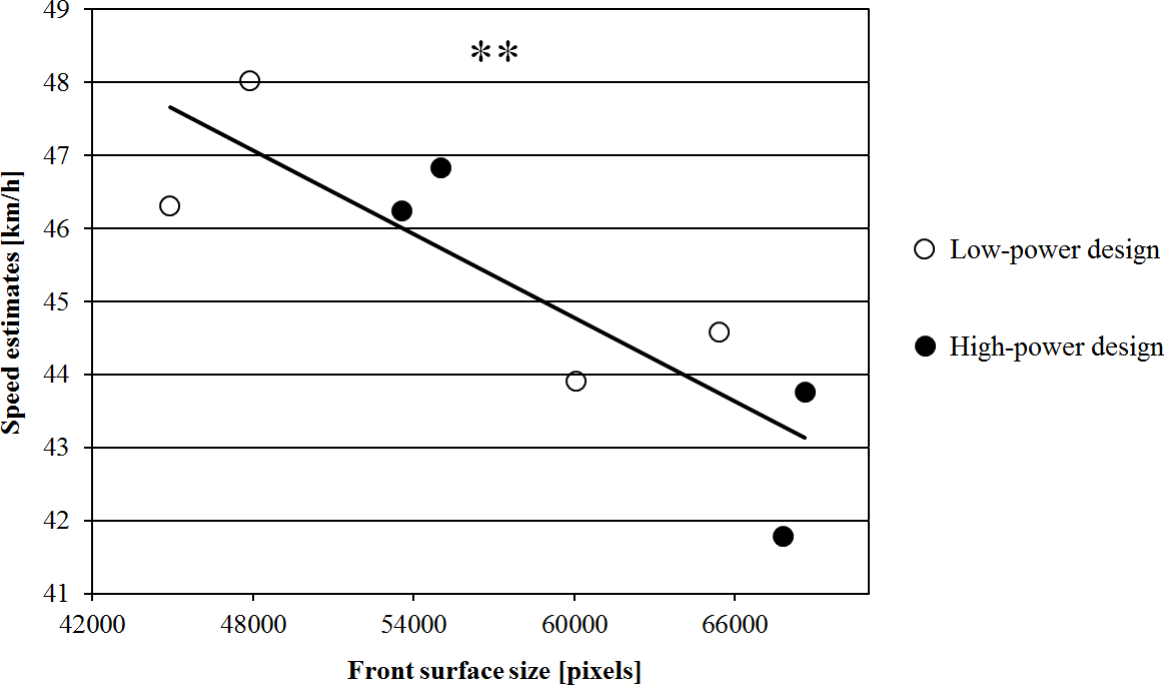
Correlation between front surface size and mean speed estimations.

Speed estimations ranged between 10 and 100 km/h (between 2.78 and 27.78 m/s; *M* = 45.2 km/h, *SD* = 13.8). A 2 (power) × 2 (position) × 2 (car size) × 3 (actual speed) repeated measures ANOVA revealed a significant main effect of power, *F*(1,59) = 13.529, *p* = .001, η_p_^2^ = .187. Low-power cars were perceived as cruising faster (*M* = 45.7 km/h, *SD* = 13.84) than high-power cars (*M* = 44.7 km/h, *SD* = 13.75). Car size was also highly significant, *F*(1,59) = 126.512, *p* < .001, η_p_^2^ = .682. Cars with a small front surface were perceived to travel with a higher speed (*M* = 46.9 km/h, *SD* = 14.1) than cars with a large front surface (*M* = 43.5 km/h, *SD* = 13.3; see Fig 4). This finding was qualified by a significant negative correlation between front surface size and mean speed estimations (*r* = –.848, *p* = .008): The bigger the front surface of the car, the lower was the speed estimate (see Fig 5). There was a main effect of position: the speed of the cars was estimated significantly lower when the participant was standing on the centre island (*M* = 44.2 km/h, *SD* = 13.3 vs. *M* = 46.2 km/h, *SD* = 14.3) compared to when standing on the pavement, *F*(1,59) = 11.104, *p* = .001, η_p_^2^ = .158. The effect of the actual speed of the vehicles also reached statistical significance, *F*(2,58) = 155.657, *p* < .001, η_p_^2^ = .843. Cars with an actual speed of 55 km/h were perceived to be significantly faster (*M* = 49.5 km/h, *SD* = 14.14) than cars that travelled with 50 km/h (*M* = 45.4 km/h, *SD* = 14.00) or 45 km/h (*M* = 40.7 km/h, *SD* = 13.26). No interaction reached statistical significance (all *p*’s > .08).

## Discussion

The aim of the present study was to investigate whether the appearance of car fronts influences pedestrians’ road-crossing behaviour and the perceived speed of passing cars. We expected that participants would cross the road earlier in front of dominant-looking cars than in front of friendly-looking cars. We also expected the perceived speed of a passing car to be influenced by its design. Our hypotheses were partially supported by the data. Power only had an influence on road-crossing behaviour in combination with car size. Only for large cars did we find that participants started to cross the road (and arrived on the other side) significantly earlier in front of dominant-looking cars compared to friendly-looking cars. For small cars we found no difference between high- and low-power design. In general, the road was crossed earlier when facing big cars than when facing small cars. Though not expected, we found that participants started crossing the road and arrived on the other side later when starting from the centre island than when starting from the pavement. Also, the speed of passing cars was perceived to be slower when observed from the central island compared to the pavement. Walking speed was not affected by power and car size but only by starting position. For the speed estimations, we found that dominant-looking cars and large cars were perceived to be passing with a lower speed than friendly-looking and small cars.

We expected that participants would be more cautious when crossing the road in front of high-power cars. We based this assumption on studies showing that car fronts are perceived in a similar way as human faces (e. g., Kloth et al. [7]; Windhager et al. [5]) and receive attributions of personality characteristics [15]. In the present study, power only had an influence on the point in time chosen to start crossing the road for large vehicles. Possibly, small cars appeared too small at the time when participants started to cross the road, so that the high/low-power car fronts could not influence the participant’s decision. On average, participants started to cross the road when the cars were 33.7 metres away and it is possible that the design effect could not unfold when the images of the cars were too small.

Piff et al. [19] found that drivers of higher-priced cars were less willing to stop at a zebra crossing than drivers of mid-priced cars. The price segment of a given car generally is associated with the dominance of a car and all high-power cars in the present study can be assigned to the high-price segment, whereas cars with low-power design can be allocated to a relatively mid-priced segment. The power effects found in the present study might hence result in part from driver attributions: Participants may have trusted the drivers of large high-power cars less than drivers of large low-power cars.

Large cars were perceived to be travelling slower than small cars. This is in line with the size-speed bias [25]: Smaller objects seem to move faster than large objects, even when the larger object is up to 57% faster than the smaller object [26]. This phenomenon is explained by the feedback of eye movements that maintain an object in the foveal region of the eye. The speed of the eye movements determines the perceived velocity of that object. Because a large object requires less effort to maintain its form in the foveal region, there are fewer smooth eye movements, resulting in an underestimation of the object’s velocity [27]. Hence, speed-overestimation can be ruled out as the reason for participants crossing the road earlier in front of large compared to small cars. In fact, the opposite is true: Although the velocity of dominant compared to friendly cars was perceived to be slower, participants started to cross the road earlier (i.e., when the car was still further away).

Somewhat surprisingly, low-power cars were perceived to be moving faster than high-power cars. It can only be speculated why this was the case. Taking the study of Piff et al. [19] into account, a possible explanation might be that participants might have expected drivers of high-power cars to be driving faster than drivers of friendly-looking cars. Participants may hence have been surprised by the relatively slow speed of cars with a dominant appearance, leading to an underestimation of the speed. Conversely participants may have anticipated friendly-looking cars to be travelling more slowly and as a result overestimated the speed of these cars.

In both blocks we found that the starting position (pavement vs. centre island) had an effect. In the road-crossing block, participants started to cross the road later when starting from the traffic island compared to the pavement. Why should participants behave in a riskier way when standing on the central refuge than on the pavement? On the one hand, it is more dangerous to stand on the refuge because it is in the middle of the road. On the other hand, on the island pedestrians might think that they are more visible to the driver. Moreover, when standing on the centre island the pedestrian’s intention to cross the road is more obvious than when standing on the pavement. Participants may also have behaved in a riskier manner because they underestimated the speed of the oncoming traffic (Block 2). An alternative explanation for the influence of position (pavement vs. centre island) might be the moving direction of the cars. Whether an object is moving from left to right or from right to left has been shown to influence speed and motion perception [28]. In a study using a representational momentum paradigm Halpern and Kelly [28] reported a larger memory bias in implied motion to the right compared to implied motion to the left. Specifically, they found that objects which were apparently moving from left to right were perceived as having moved forward more than objects moving from right to left. A similar direction bias might be responsible for the effects found in the present study: when standing on the pavement cars were passing from left to right while they were passing from right to left when standing on the centre island. Coincidently, cars were perceived as travelling faster when moving from the left to right than when moving from right to left. It would be interesting to replicate this study in left-hand driving countries such as for example the UK to clarify whether our findings can be attributed to a pure position effect (island vs. pavement) or whether they are the result of a direction bias (movement from left to right vs. movement from right to left).

In the speed-estimation block, we found that participants were able to recognize the slight differences in speed (45/50/55 km/h or 12.5/13.89/15.28 m/s). In general, estimates were too low, possibly due to the sparse virtual environment, which hardly provided reference points. Furthermore, the cars approached almost directly towards the observer, offering only marginal motion parallax. This may have led to participants being highly unreliable in speed perception, with their speed estimations ranging from 10 to 100 km/h (2.78 to 27.78 m/s).

Even though virtual reality environments have many advantages in studying pedestrian behaviour and have been shown to be a valid instrument to experimentally measure the behaviour of pedestrians [22], this study has some limitations. The artificial appearance of the virtual environment may make the situation as a whole slightly unrealistic. We note however that the 3D models of the cars used in the present study received highly comparable ratings as those in the study of Windhager et al. [15]: high-power cars were rated as being more arrogant, more extraverted, less surprised, less afraid-looking, angrier, less sad, less agreeable, more adult, more dominant, more hostile, and more masculine than low-power cars. Nevertheless, the present VR design might be slightly disadvantageous in measuring effects of car design on behaviour because passing vehicles are only visible in full size for a relatively short time. Future studies could use lower speeds so that the power can take effect over a longer time. Finally, we note that despite the advantages of virtual reality, this experiment allows only for an estimation of what might happen in real world situations. Future field studies are needed to confirm whether our experimental findings can be translated to real world behaviour.

To our knowledge, this is the first study testing the effect of car front design on pedestrian behaviour. By using existing car models that commonly run on European roads the study has high ecological validity. We found that the combination of large car size and dominant appearance lead people to cross the road earlier. Our findings suggest that large vehicles with a dominant appearance may cause fewer pedestrian accidents than large vehicles with a friendly appearance. Hence, road safety may benefit from vehicles with large size and dominant design, causing other road users to behave more cautiously. This might be an important implication as the automobile industry is facing a fundamental change, moving from combustion engine to electromobility. As pointed out by Purucker et al. [14], the design of today’s car fronts conform to practical engineering needs such as the radiator grille. The authors raise the question whether anthropomorphic designs in car fronts might disappear when technological requirements change. In line with Purucker et al. [14], our results suggest that even without a functional need, anthropomorphic design in car fronts may be important to improve road safety of pedestrians.

To conclude, the present study suggests that car design can influence pedestrian road crossing behaviour. We found that participants started to cross the road earlier in front of large compared to small cars. For cars that were large in size, we additionally found an effect of power: participants crossed the road earlier in front of large powerful looking cars compared to large friendly-looking cars. These findings cannot be explained by biased speed estimations because participants perceived large and high-power cars to be travelling slower than small and low-power cars. Car design hence had an influence on road-crossing behaviour irrespective of perceived speed of the vehicle.

## Supporting Information

S1 File Data of road-crossing and speed-estimation task. (SAV)
